# Uncommon Culprit, Familiar Foe: A Case of Septic Thrombophlebitis of the Internal Jugular Vein Triggered by Klebsiella Bacteremia

**DOI:** 10.7759/cureus.56716

**Published:** 2024-03-22

**Authors:** Ekrem Yetiskul, Alaukika Agarwal, Salman Khan, Faris Qaqish, Taqi A Rizvi, Hamzah Qandil, Neville Mobarakai

**Affiliations:** 1 Internal Medicine, Northwell Health - Staten Island University Hospital, Staten Island, USA; 2 Infectious Disease, Northwell Health - Staten Island University Hospital, Staten Island, USA

**Keywords:** central venous catheter (cvc), intravascular device, bacteremia, klebsiella, septic thrombophlebitis

## Abstract

Septic thrombophlebitis of the internal jugular vein is characterized as Lemierre syndrome. Patients typically present with sore throat and fever and may present with a tender neck mass due to thrombophlebitis of the internal jugular vein. We present the case of a 57-year-old male with neck pain, fever, chills, and headaches who was diagnosed with internal jugular vein septic thrombophlebitis associated with catheter-related introduction of bacteria.

## Introduction

Septic thrombophlebitis is characterized by venous wall inflammation and thrombosis associated with prolonged bacteremia. It is usually encountered in patients with defined underlying conditions such as the presence of an intravascular device, burns, and malignancy [1]. The treatment principles for septic thrombophlebitis are not well defined but commonly involve source control, removing the intravascular device and/or surgical debridement of the foci of infection, and administering appropriate antimicrobial therapy, chemotherapy, and anticoagulation [1]. Septic thrombophlebitis of the internal jugular vein has been classically associated with oropharyngeal infections but very rarely with central venous catheters (CVCs) [2]. Catheters may be the source for the introduction of bacteria that leads to thrombosis and infection. This is distinct from infection typically arising from the oral intestinal mucosa known as Lemierre syndrome, which has an incidence of approximately 3.6 cases per million individuals [3]. *Fusobacterium necrophorum* has been well-established as the classical pathogen associated with Lemierre syndrome [4]. In contrast, the order of selected pathogens associated with causing central line-associated bloodstream infection (CLABSI) is as follows: predominantly, gram-positive organisms (including coagulase-negative Staphylococci at 34.1%, Enterococci at 16%, and *Staphylococcus aureus* at 9.9%) emerge as the most prevalent, succeeded by gram-negative organisms (with *Klebsiella *at 5.8%, *Enterobacter *at 3.9%, *Pseudomonas *at 3.1%, *Escherichia coli* at 2.7%, and *Acinetobacter *at 2.2%). *Candida *species contribute to 11.8% of cases, while other pathogens collectively account for 10.5% [5]. Although *Klebsiella *species is known to be a possible pathogen in CLABSI and an extremely rare cause of Lemierre syndrome, data regarding the association between internal jugular vein septic thrombophlebitis and CLABSI caused by *Klebsiella* species is limited [5,6]. We present the case of a 57-year-old male with a history of Stiff-person syndrome actively undergoing plasmapheresis (PLEX) via tunneled dialysis catheter who developed right internal jugular vein thrombophlebitis with concurrent *Klebsiella *bacteremia.

## Case presentation

We present the case of a 57-year-old male with a past medical history of hypertension, hyperlipidemia, benign prostatic hyperplasia, a transient ischemic attack, and stiff-person syndrome who was admitted for inpatient PLEX. The patient had a tunneled dialysis catheter placed in his right internal jugular vein for this purpose. The patient completed a nine-day course of PLEX, and the catheter was consequently removed and the patient was discharged. The patient returned home, but he began to experience “throbbing” neck pain the next day, which was worse with movement and accompanied by fevers, chills, and intermittent headaches. This resulted in the patient presenting to the emergency department again four days after his initial discharge. He denied dizziness, vision loss, dyspnea, dysphagia, nausea, and vomiting.

In the emergency department, the patient’s vital signs included a temperature of 37.6°C (99.7°F), a heart rate of 138 beats per minute, a blood pressure of 137/83 mmHg, a respiratory rate of 18 breaths per minute, and oxygen saturation level of 99% on ambient room air. Physical examination revealed right-sided neck tenderness to palpation, a palpable cord, erythema, and swelling.

**Figure 1:**
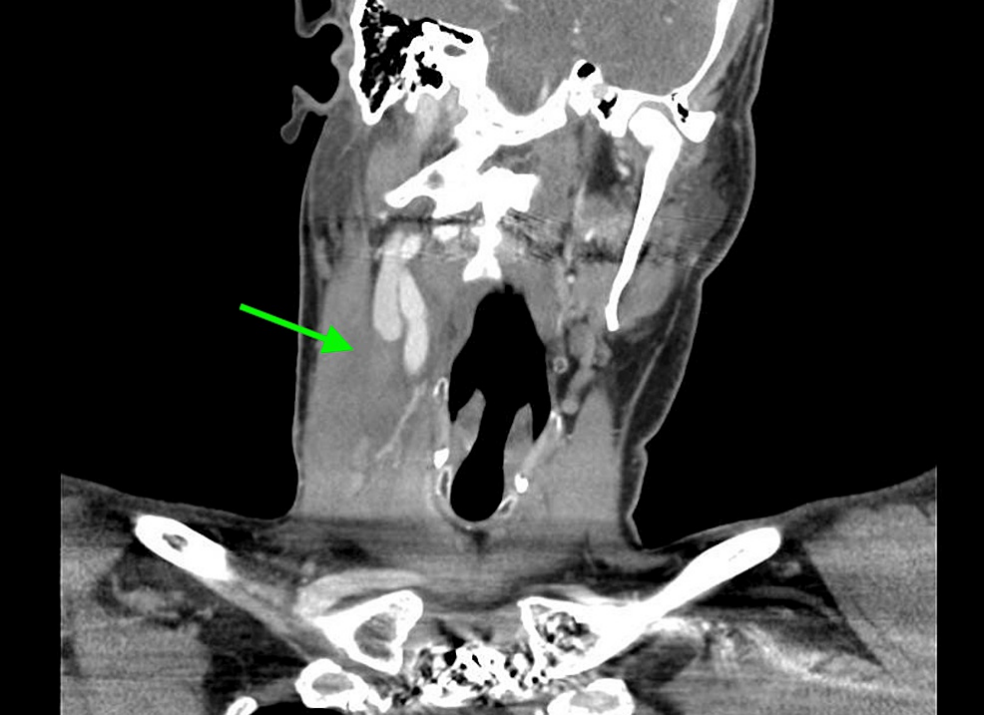
Coronal section obtained from CT of the neck with intravenous contrast demonstrates a sudden loss of contrast opacification in the right internal jugular vein, accompanied by surrounding stranding and edema, suggesting thrombophlebitis with no discernible fluid collection amenable to drainage (green arrow).

**Figure 2:**
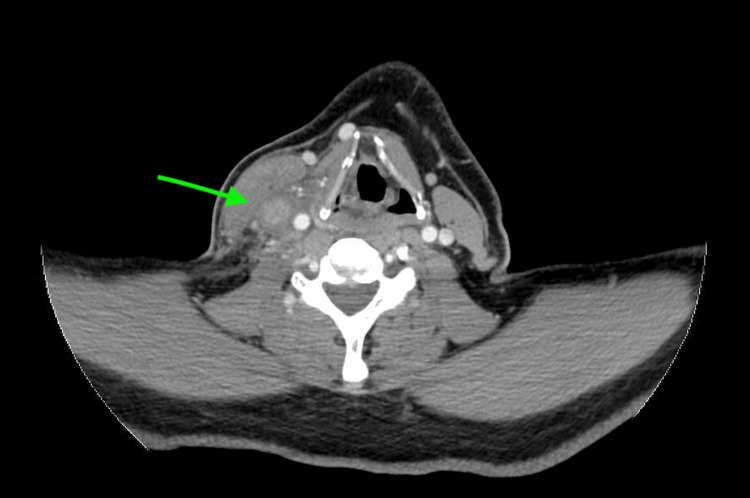
Axial section obtained from CT of the neck with intravenous contrast demonstrates a sudden loss of contrast opacification in the right internal jugular vein, accompanied by surrounding stranding and edema, suggesting thrombophlebitis with no discernible fluid collection amenable to drainage (green arrow).

Laboratory findings in the emergency department revealed a white blood cell count of 8.06 K/µL (reference range: 4.8-10.8 K/µL). All other laboratory findings were within normal limits. Considering the patient’s recent history of right internal jugular venous catheter thrombosis, a computed tomography scan of the neck with intravenous contrast was performed. The imaging revealed a sudden loss of contrast opacification in the right internal jugular vein, accompanied by surrounding stranding and edema, suggesting thrombophlebitis with no discernible fluid collection amenable to drainage (Figures 1, 2).

Given the presence of right internal jugular thrombophlebitis, therapeutic rivaroxaban and ceftriaxone were started. Blood cultures drawn on admission yielded growth of various *Klebsiella *group species, including *K. pneumoniae*, *K. quasipneumoniae*, and *K. variicola*. A transthoracic echocardiogram was unremarkable for any findings suggestive of infective endocarditis. Over the next five days, the patient’s neck pain improved. He was discharged with instructions to continue anticoagulation therapy with rivaroxaban for three months, complete two weeks of intravenous ceftriaxone, and then start oral levofloxacin for two weeks.

## Discussion

The use of CVCs is a widespread practice in medicine, but these devices are well-known for increasing susceptibility to infections. The source of infection in CLABSI is the introduction of a foreign catheter into the bloodstream, which acts as a nidus for bacteria. However, Lemierre syndrome arises from an endemic bacteria in the oral flora. The source of these infections accounts for the difference in these two pathologies, with similar treatment approaches involving antimicrobial therapy and anticoagulation.

In this case, the patient, a 57-year-old male actively undergoing PLEX for stiff-person syndrome via a tunneled dialysis catheter, developed right internal jugular vein thrombophlebitis with concurrent *Klebsiella* bacteremia. This case highlights one of the severe complications of CLABSI, leading to septic thrombophlebitis of the internal jugular vein caused by *Klebsiella* species.

*Klebsiella* is a gram-negative bacteria that has been shown to cause inflammation and thrombosis in the context of several pathologies [7]. *K. pneumoniae* has been associated with Lemierre syndrome, an infection in the oropharynx with thrombosis of the internal jugular vein [4]. This was highlighted in a case report of a 45-year-old woman who presented with left neck swelling and septicemia and was consequently diagnosed with Lemierre syndrome. This bacteria is also established as a causative pathogen in septic pulmonary embolism, presented in various case reports, including in an intravenous drug user and a patient with a previous atrioseptal defect operation [8,9]. Widespread septic emboli are a common feature of Lemierre syndrome with a case report describing a 19-year-old found to have *K. pneumoniae *septic thrombophlebitis complicated by epidural abscess [6].

There is also an unusual case with six episodes of recurrent *K. pneumoniae *sepsis within a six-month duration in an adolescent who underwent hematopoietic stem cell transplantation for acute lymphoblastic leukemia [10]. This patient required a CVC for chemotherapy, predisposing him to CLABSI, malfunction, and thrombosis. In contrast, the microbial profile of the blood cultures in our patient revealed the growth of various *Klebsiella* group species, including *K. pneumoniae, K. quasipneumoniae, *and *K. variicola*. This finding is noteworthy as *Klebsiella* species has an incidence of 5.8% of CLABSI [5], and species such as *K. quasipneumoniae* and *K. variicola* have not been explored in thrombophlebitis.

The multifaceted approach involved in managing septic thrombophlebitis also requires careful consideration. This includes the need for source control, which requires the removal of the CVC. In our case, this patient was undergoing plasmapheresis, which emphasizes the importance of timely recognition and intervention for optimal outcomes. Initiation of therapeutic anticoagulation with rivaroxaban was used to manage the thrombotic component of the condition. Broad-spectrum antibiotic therapy with ceftriaxone was also administered to target the causative *Klebsiella *species. In most cases, patients are treated empirically with broad-spectrum antibiotics and then de-escalated to more narrow-spectrum antibiotics based on bacterial culture and sensitivities. The treatment approach is generalized based on individual patient factors, including severity of infection, and extent of thrombosis. Patients may frequently require follow-up with imaging studies to assess for resolution or progression of thrombosis. The patient demonstrated clinical improvement over the hospital course with prompt and appropriate treatment. This highlights the need to tailor the treatment regimen toward individual patient approaches. There are no specific guidelines available regarding the treatment of this condition, and the physician is required to exercise his or her judgment to craft a treatment strategy.

## Conclusions

This case characterizes a need for a comprehensive understanding of complications associated with CVCs, focusing on preventive strategies for CLABSI. For a favorable outcome, healthcare providers should remain vigilant in their surveillance for catheter-related complications, especially for patients with underlying autoimmune conditions due to their increased susceptibility to infection, increased risk of thrombosis, as seen in systemic lupus erythematosus, and potential drug interactions with immunosuppressive therapy.
